# Indoor grown cannabis yield increased proportionally with light intensity, but ultraviolet radiation did not affect yield or cannabinoid content

**DOI:** 10.3389/fpls.2022.974018

**Published:** 2022-09-27

**Authors:** David Llewellyn, Scott Golem, Elizabeth Foley, Steve Dinka, A. Maxwell P. Jones, Youbin Zheng

**Affiliations:** ^1^School of Environmental Science, University of Guelph, Guelph, ON, Canada; ^2^HEXO Corp., Gatineau, QC, Canada; ^3^Department of Plant Agriculture, University of Guelph, Guelph, ON, Canada

**Keywords:** ultraviolet (UV), secondary metabolites, light emitting diode (LED), cannabinoid, eustress, harvest index

## Abstract

Cannabis (*Cannabis sativa*) flourishes under high light intensities (LI); making it an expensive commodity to grow in controlled environments, despite its high market value. It is commonly believed that cannabis secondary metabolite levels may be enhanced both by increasing LI and exposure to ultraviolet radiation (UV). However, the sparse scientific evidence is insufficient to guide cultivators for optimizing their lighting protocols. We explored the effects of LI and UV exposure on yield and secondary metabolite composition of a high Δ^9^-tetrahydrocannabinol (THC) cannabis cultivar ‘Meridian’. Plants were grown under short day conditions for 45 days under average canopy photosynthetic photon flux densities (PPFD, 400–700 nm) of 600, 800, and 1,000 μmol m^–2^ s^–1^, provided by light emitting diodes (LEDs). Plants exposed to UV had PPFD of 600 μmol m^–2^ s^–1^ plus either (1) UVA; 50 μmol m^–2^ s^–1^ of UVA (315–400 nm) from 385 nm peak LEDs from 06:30 to 18:30 HR for 45 days or (2) UVA + UVB; a photon flux ratio of ≈1:1 of UVA and UVB (280–315 nm) from a fluorescent source at a photon flux density of 3.0 μmol m^–2^ s^–1^, provided daily from 13:30 to 18:30 HR during the last 20 days of the trial. All aboveground biomass metrics were 1.3–1.5 times higher in the highest vs. lowest PPFD treatments, except inflorescence dry weight – the most economically relevant parameter – which was 1.6 times higher. Plants in the highest vs. lowest PPFD treatment also allocated relatively more biomass to inflorescence tissues with a 7% higher harvest index. There were no UV treatment effects on aboveground biomass metrics. There were also no intensity or UV treatment effects on inflorescence cannabinoid concentrations. Sugar leaves (i.e., small leaves associated with inflorescences) of plants in the UVA + UVB treatment had ≈30% higher THC concentrations; however, UV did not have any effect on the total THC in thesefoliar tissues. Overall, high PPFD levels can substantially increase cannabis yield, but we found no commercially relevant benefits of adding UV to indoor cannabis production.

## Introduction

Drug-type *Cannabis sativa* (hereafter: cannabis) is one of the highest-value crops that are commercially grown in indoor environments, where electric lighting is the principal source of photosynthetically active radiation (PAR; 400–700 nm; Zheng and Llewellyn, 2022). Given cannabis’ high market value and exceptional tolerance of high light intensity (LI) (Chandra et al., 2008; Rodriguez-Morrison et al., 2021a), canopy level photosynthetic photon flux densities (PPFD) can be several-fold higher than for many other indoor-grown commodities (Bilodeau et al., 2019). In order to optimize profitability, the concomitant premiums in energy and lighting (and related) infrastructure costs must be carefully considered as offsets to any potential increases in yield and quality that may be achieved under higher LI. A major component of this balancing process is the development of response models of cannabis yield and quality to a commercially relevant range of LIs. The photosynthesis, growth and yield models to LI presented by Rodriguez-Morrison et al. (2021a) on a cannabidiol (CBD) dominant cannabis cultivar illustrated cannabis’ immense capacity to convert PAR into marketable biomass. Their results showed that cannabis inflorescence yield increased linearly with increasing canopy PPFD up to 1,800 μmol m^–2^ s^–1^ along with minor increases in secondary metabolite concentrations (notably no LI effects on CBD concentrations). Notwithstanding their results, the LI responses of cultivars with different growth habits and secondary metabolite profiles [e.g., Δ^9^-tetrahydrocannabinol (THC) dominant] is warranted to round out our understanding of cannabis LI responses.

The efficient production of marketable biomass (i.e., mature, unfertilized female inflorescences) is clearly of foremost importance in cannabis production. However, indoor-grown cannabis is a highly specialized crop, in that the major product of interest is not simple biomass but the secondary metabolites. These are predominantly cannabinoids and terpenes, which are chiefly associated with the inflorescence tissues from unfertilized female plants (hereafter: inflorescence) (Potter, 2009; Small, 2017; Livingston et al., 2020). In modern drug-type cannabis genotypes, these secondary metabolites can comprise ≥25% of the total biomass in mature inflorescence tissues and this is one of the primary metrics determining marketability (Dujourdy and Besacier, 2017; Jikomes and Zoorob, 2018; Cash et al., 2020).

Cannabinoids may have photoprotective roles in cannabis ecology, with some (sparse) scientific evidence that light stress from either high LI or spectral manipulations can alter the metabolomic composition (see: Magagnini et al., 2018 for a review). Further, it has been posited that the ultraviolet (UV) absorption properties of some cannabinoids may represent an ecological justification (i.e., as photoprotective elements) for why high concentrations of some cannabinoids are associated with inflorescence tissues. In particular, some older studies alluded to possible links between UV exposure and THC content (Fairbairn and Liebmann, 1974; Pate, 1983; Lydon et al., 1987) in indoor-grown cannabis. However, no mechanism for upregulating THC vs. other cannabinoids under UV exposure has been elucidated (Pate, 1994). Further, UV absorption of THC does not confer a clear ecological advantage relative to other major cannabinoids, which have similar [e.g., cannabidiol (CBD)] or greater [e.g., cannabichromene (CBC) and cannabinol (CBN)] UV absorption than THC (Pate, 1994; Hazekamp et al., 2005; de Backer et al., 2009). Despite a lack of contemporary published scientific studies on the effects of UV exposure on cannabinoid content (Magagnini et al., 2018), there is a popular belief that UV exposure can substantially enhance cannabinoid content – particularly THC – in inflorescence tissues in modern cannabis genotypes. Genotypic predisposition to producing THC is also an important consideration since inflorescence THC content may be many times higher in modern vs. older cannabis genotypes (Dujourdy and Besacier, 2017). Therefore, genetic factors may play a more significant role in altering inflorescence THC content than environmental stresses such as UV exposure.

The strongest links between UV radiation and cannabinoid content relate to ultraviolet-B (UVB, 280–315 nm), however, radiation from the ultraviolet-A (UVA, 315–400 nm) and shorter wavelengths in the blue (400–500 nm) wavebands have also been implicated in altering the cannabis inflorescence chemical composition (Magagnini et al., 2018; Bilodeau et al., 2019) and mediating cellular repair provoked by UVB damage in other species (Krizek, 2004), sometimes called photoreactivation (Gill et al., 2015). Rodriguez-Morrison et al. (2021b) found only deleterious effects on cannabis morphology, physiology, yield, and quality when two chemotype II cannabis cultivars were exposed to various levels of short wavelength UVB provided by LEDs with a peak wavelength of 287 nm. However, since very little solar UV with wavelengths below 295 nm reaches the earth’s surface (Green et al., 1974; Flint and Caldwell, 1996), cannabis plants may have no adaptive or acclimative mechanisms for coping with periodic exposure to such short wavelength radiation. Further, the relative impacts of wavelength on plant growth increases exponentially with decreasing wavelength within the narrow UVB waveband, spanning almost two orders of magnitude over just 35 nm (Flint and Caldwell, 2003). Therefore, longer UV wavelengths and shorter exposure periods may be able to provoke the desired upregulation of secondary metabolite production with minimized negative responses on plant growth and yield.

The objectives of this study were to investigate the impacts of increasing LI and exposure to UVA and UVB on the yield and quality of mature female inflorescences in an indoor grown, high-THC cannabis genotype. The experiment was conducted in an indoor environment with three LI treatments and two UV treatments where light emitting diodes (LEDs) were the sole source of PAR and UV treatments were provided using both LED and UV fluorescent lighting technologies.

## Materials and methods

The experiment was conducted in a commercial cannabis production facility in Southern Ontario, Canada. Three enclosures (5.9 m × 4.1 m × 2.7 m) were constructed within a common production area. Each enclosure consisted of two benches (5.9 m × 1.8 m) that were separated by 0.5 m and encompassed with “panda film” (Vivosun, City of Industry, CA, USA), black side facing inward, to eliminate light contamination external to the enclosures. Each enclosure contained six 0.63 m^2^ plots, with a lateral separation of ≥0.65 m between plots. Air temperature and relative humidity (RH) were recorded every 300 s using data loggers (HOBO MX2301A; Onset Computer Corporation, Bourne, MA, USA) centered in each enclosure at the same elevation as the light fixtures level. Across the three enclosures, the daytime temperature and RH were (mean ± SD) 26 ± 1.2°C and 40 ± 6.9%, respectively, and nighttime temperature and RH were 22 ± 1.9°C and 47 ± 3.9%, respectively. No supplemental CO_2_ was provided during this trial.

### Lighting treatments

**FIGURE 1:**
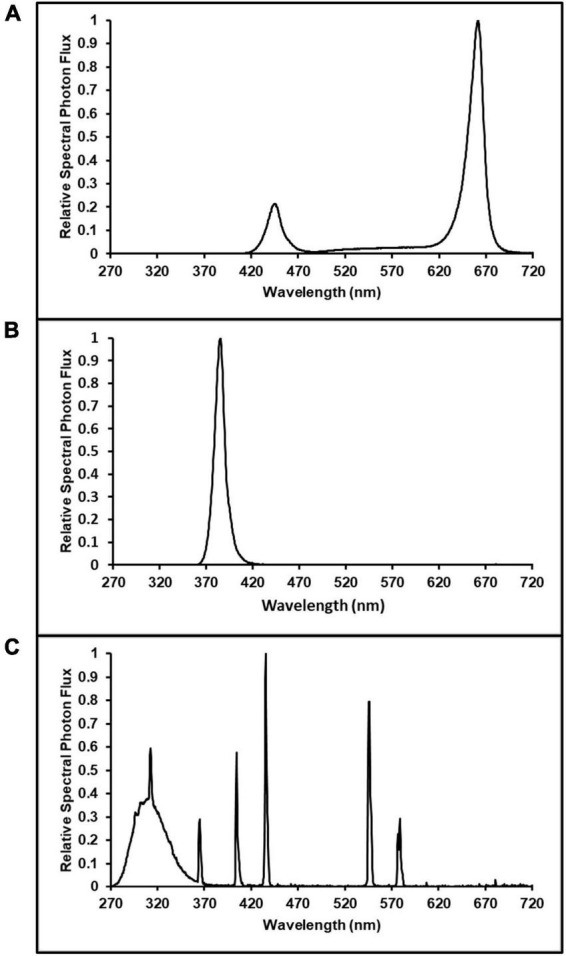
Spectrum distributions of: **(A)** Lumigrow Toplight LED source of photosynthetically active radiation; used in all ultraviolet (UV, 280–400 nm) spectrum and intensity treatments, **(B)** 385 nm peak UVA LEDs; used in UVA treatment (photon flux density of 50 μmol m^–2^ s^–1^), and **(C)** broad-band fluorescent UV; used in the UVA + UVB treatment (photon flux density of 3 μmol m^–2^ s^–1^).

Pairs of LED bars (Toplight-Targeted Spectrum; LumiGrow, Emeryville, CA, USA) were spaced 0.4 m apart, on-center, over each plot. One plot in each enclosure had an additional pair of Toplight LED bars, evenly spaced between the first pair of LED bars, to facilitate higher light intensities in this plot. These fixtures were comprised of a combination of blue, phosphor-converted white (5,000 K), and red LEDs. The blue and red LEDs had peak wavelengths [± half-width at half maximum (HWHM)] of 445 ± 8.5 nm and 665 ± 8.0 nm, respectively. When all channels were operated at maximum intensity, the native spectrum (Figure 1A) of the Toplight fixtures had a photon flux ratio of blue (B, 400–500 nm), green (G, 500–600 nm) and red (R, 600–700 nm) of B17:G7:R76. This spectrum was maintained in all cases where dimming was used to reduce intensity. Initial fixture hang heights were 0.9 m above the bench. The UVA spectrum (Figure 1B) was provided by custom-made LED bars (size 0.05 m × 0.6 m; Yunustech, Mississauga, ON, Canada) which had a peak wavelength (± HWHM) of 385 ± 5.5 nm. In one plot in each enclosure, two UVA LED bars were centered 0.24 m apart, at the same height of the LED Toplight fixtures. The UVA + UVB spectrum (Figure 1C) was provided by a broad-band fluorescent lighting technology (SolarSystem UVB, California LightWorks, Canoga Park, CA, USA). A single SolarSystem UVB fixture was centered over one plot in each enclosure, positioned at the same height as the LED Toplight fixtures.

**FIGURE 2:**
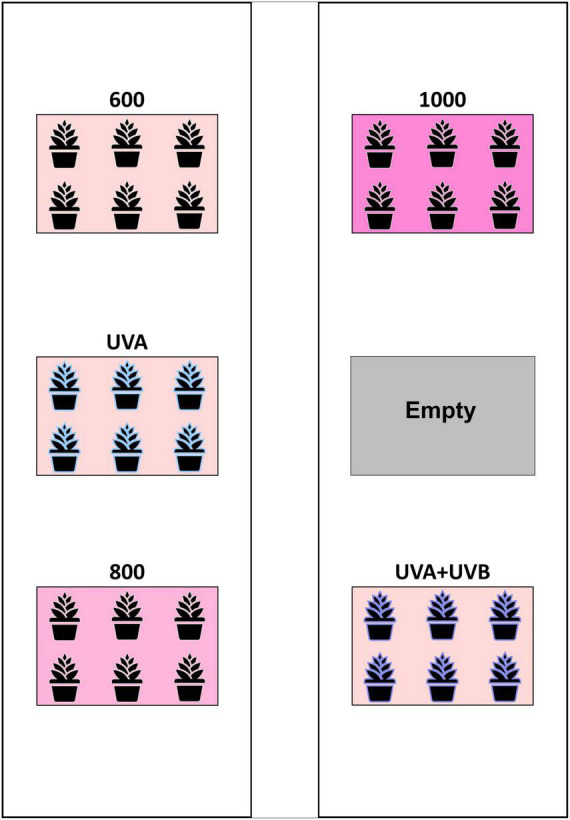
Schematic of the layout of treatment plots within each enclosure. The arrangement of the five treatments was randomized within the six plot locations in each enclosure (i.e., block) with the sixth plot in each block remaining empty.

The experiment was arranged as a randomized complete block design (RCBD) with three LI and two UV-spectrum treatments and three concurrent replications (blocks). In each block (i.e., the enclosures), the three intensity treatment plots targeted canopy-level PPFDs of 600 (control), 800, and 1,000 μmol m^–2^ s^–1^, respectively. The UV-spectrum treatments had targeted canopy-level PPFDs of 600 μmol m^–2^ s^–1^ plus UV photon flux densities (PFD) of 50 and 3.0 μmol m^–2^ s^–1^ for the UVA and UVA + UVB treatments, respectively. In each enclosure (i.e., block), the locations of the five treatment plots were randomly arranged among the six bench positions, with one plot in each block remaining empty (Figure 2). All of the Toplight and UVA fixtures had a photoperiod of 12 h (06:30 to 18:30 HR). The SolarSystem UVB fixtures in UVA + UVB treatment plots had a 5-h photoperiod (13:30 to 18:30 HR) and were only operated during the last 20 days of the trial. The spectra were evaluated using a radiometrically-calibrated spectrometer (XR-Flame-S; Ocean Optics, Dunedin, FL, USA) coupled to a CC3 cosine-corrector attached to a 1.9 m × 400 μm UV-Vis optical fiber. A MS Excel tool developed by Mah et al. (2019) was used to integrate spectral irradiance data into PPFD, UV-PFD, and compute biologically-effective UV-PFD and radiant flux density (in the UV treatment plots). Intensities of the Toplight LEDs were modified using the lighting control software (smartPAR; LumiGrow) to achieve the prescribed intensity and spectra. The UV spectra and intensity levels in the UV treatment plots were characterized with the Toplight LEDs turned off. The UV intensity in the UVA treatment was adjusted using constant current dimmers. The UV intensity in the UVA + UVB treatment was adjusted using aluminum neutral density screen affixed to underside of the SolarSystem UVB fixture. The dominant peak in the UVA + UVB spectrum spanned from ≈275 to 380 nm, with additional narrow-band peaks at 312, 365, 404, 435, 545, and 579 nm (Figure 1C). On a photon-flux basis, the spectrum had a ratio of UVB to UVA of 1.07 (i.e., almost equal photon flux levels of UVB and UVA). Flux density integrals of the UV spectrum treatments, based on both raw flux density data and on conversions according to the Biological Spectral Weighting Function (BSWF; Flint and Caldwell, 2003), are presented in Table 1.

**TABLE 1:** Exposure periods, instantaneous and daily integrated ultraviolet (UV, 280–400 nm) flux densities at canopy level of the UVA and UVA + UVB spectrum treatments based on both raw intensities and converted using the Biological Spectral Weighting Function (BSWF; Flint and Caldwell, 2003), and daily light integrals (DLI) of photosynthetically active radiation (400–700 nm).

Parameter	Units	Spectrum treatment		Rodriguez-Morrison et al. (2021b)	Lydon et al. (1987)
		UVA	UVA + UVB		
Daily UV exposure time	h	12	5	3.5	6
UV treatment period	Day	45	20	60	40
**Raw UV flux density**					
UV PFD^z^	μmol⋅m^–2^⋅s^–1^	50	3.0	≤0.8	
UV RFD^y^	W⋅m^–2^	16	1.1		
Daily dose	kJ⋅m^–2^⋅day^–1^	670	20		
**Biological Spectral Weighting Function**					
UV PFD	μmol_(B)_⋅m^–2^⋅s^–1^	0.67	1.6	≤2.6	
UV RFD	W_(B)_⋅m^–2^	0.21	0.59		
Daily dose	kJ_(B)_⋅m^–2^day^–1^	9.1	10	≤13	≤13.4
Total dose	kJ_(B)_⋅m^–2^	410	200	≤780	≤536
**Photosynthetically Active Radiation**					
DLI	mol⋅m^–2^⋅day^–1^	25.9	25.9	17.6	13.5

Also included are the published maximum values for the known parameters from Lydon et al. (1987) and Rodriguez-Morrison et al. (2021b); the UV dose in Lydon et al. (1987) was calculated using an earlier version of the BSWF (Caldwell, 1971) that only integrates UV wavelengths between ≈ 275 and 315 nm).

^y^Radiant flux density.

^z^Photon flux density.

Following spectrum characterization, the canopy-level PPFD of each plant was measured and recorded, twice weekly, using a handheld light sensor (LI-180; LI-COR Biosciences, Lincoln, NE, USA) that was spectrum-matched to the PPFD readings of the XR-Flame-S spectrometer under the PAR spectrum in Figure 1A. Light fixture hang-heights were adjusted accordingly to ensure the targeted LI levels in each plot were maintained as the plants grew.

### Plant cultivation

Uniform rooted cuttings of the clonal cannabis genotype ‘Meridian’ were transplanted into rockwool cubes (0.15 m × 0.15 m × 0.15 m; Grodan, Milton, ON, Canada) and grown using the Toplight-Targeted Spectrum (LumiGrow) fixture, as described above. Transplants were grown for 21 days under PPFD of ≈600 μmol m^–2^ s^–1^ and were maintained in a vegetative state using a 16-h photoperiod (06:30 to 22:30 HR). The transplants selected for the trial were trimmed to uniform height of 33.2 ± 2.62 cm (mean ± SD, *n* = 90). In each plot, the trimmed transplants were arranged in two rows of three, with plants spaced 45 cm apart, on center (i.e., planting density of ≈10 plants/m^2^). The PAR photoperiod was set to 12-h in every plot to induce flowering, and plants were grown for an additional 45 days under their respective lighting treatments and then harvested. Throughout the vegetative and flowering stages, plants were drip-irrigated twice daily at 2 L h^–1^ for 540 s, such that each plant received ≈0.6 L day^–1^. The nutrient solution was comprised of Dutch Nutrients Gro A and Gro B (Homegrown Hydroponics, Toronto, ON, Canada) at a rate of 5 mL L^–1^ in rainwater, resulting in an EC of ≈1.75 dS m^–1^ and pH of ≈5.6.

### Growth and yield parameters

At harvest, the stems were cut at growing medium level and the inflorescences were hand-trimmed from each plant. Selected leaves from the upper canopy of each plant in the control, UVA, and UVA + UVB treatments were photographed individually. The fresh weight of inflorescence (FW_*f*_) and non-floral (FW_*nf*_) aboveground tissues (i.e., stems and leaves) were recorded using a digital scale (AX622N/E Adventure Precision Balance; OHAUS Corporation, Parsippany, NJ, USA). The plants were harvested and processed one at a time and FW of aboveground tissues were taken within 5 min of each plant being cut. The separated aboveground tissues from one randomly selected plant in each plot (i.e., three plants per treatment) were oven-dried to constant weight at 65°C and re-weighed (AX622N/E Adventure Precision Balance; OHAUS Corporation) to determine water content of the respective tissues. After determining there were no treatment effects on tissue water content, the dry weight of inflorescence (DW_f_) and non-floral (DW_nf_) aboveground tissues were calculated for each plant using the average water content for the respective tissues. Harvest index (HI) for each plant was calculated using the following formula: HI = DW_f_/(DW_f_ + DW_nf_). The entire inflorescence tissue from one randomly selected plant in each LI treatment plot were spread out in a single layer on perforated drying trays and air dried at (mean ± SD) 19 ± 1.8°C and 51 ± 9.2 RH% for 5 days (i.e., final moisture content of ≈10–15%). Drying room air temperature and RH were recorded every 300 s using a data logger (HOBO MX1102A, Onset) centered in the drying room. Once dry, the inflorescence material from each plant was homogenized and composite ≈5 g samples were collected and submitted to a 3rd-party lab (RPC; Fredericton, NB, Canada) for analysis of cannabinoid, terpene and water content. The following cannabinoids were tested for using methanol/chloroform solvent extraction and high performance liquid chromatography (HPLC) separation with variable wavelength UV detection (VWD): cannabigerol (CBG), cannabigerolic acid (CBGA), total equivalent cannabigerol (T-CBG), cannabidiol (CBD), cannabidiolic acid (CBDA), total equivalent cannabidiol (T-CBD), Δ^9^-tetrahydrocannabinol (THC), Δ^9^-tetrahydrocannabinolic acid (THCA), total equivalent Δ^9^-tetrahydrocannabinol (T-THC), Δ^8^-tetrahydrocannabinol (D8THC), and cannabinol (CBN). The following terpenes were tested for using hexane/ethanol solvent extraction and gas chromatography (GC) separation with mass spectrometry detection (MSD): alpha pinene, beta pinene, myrcene, limonene, terpinolene, linalool, terpineol, caryophyllene, humulene, 3-carene, cis-ocimene, eucalyptol, trans-ocimene, fenchol, borneol, valencene, cis-nerolidol, trans-nerolidol, guaiol, alpha-bisabolol, and sabinene. In addition, the foliar tissues that were trimmed from the upper inflorescences (commonly called “sugar leaves” in the cannabis production industry) from two randomly selected plants from each control, UVA and, UVA + UVB treatment plot were spread out in a single layer on perforated drying trays and air dried for 5 days. Approximately 1 g of dried sugar leaves from each sample (18 samples total) were submitted to the internal lab (HEXO; Gatineau, QC, Canada) for analysis of content of the following cannabinoids: cannabichromene (CBC), CBD, CBDA, T-CBD, CBG, CBGA, T-CBG, CBN, THC, THCA, T-THC and D8THC. Analysis was done using acetonitrile solvent extraction, ultra performance liquid chromatography (UPLC) separation and VWD were extracted in acetonitrile and analyzed as per Layton and Aubin (2019). All secondary metabolite concentrations are expressed as mg g^–1^ of dry biomass, with tissue water contents determined by drying to constant weight.

### Data processing

All measured parameters were analyzed with JMP (version 10; SAS Institute Inc., Cary, NC, USA) with means separation using Tukey’s honestly significant difference test (*P* ≤ 0.05). In all cases, treatment means ± SE are presented with treatment effects denote by different lowercase letters following the means.

## Results

As LI increased from 600 to 1,000 μmol m^–2^ s^–1^, FW_f_ and DW_f_ increased by 1.5 and 1.6, times, respectively (Table 2). Compared to the 600 μmol m^–2^ s^–1^ control treatment, there were no UV spectrum treatment effects on any aboveground biomass metrics.

**TABLE 2:** Per-plant fresh weight of inflorescence (FW_f_) and non-floral aboveground (FW_nf_) tissues, dry weight of inflorescence (DW_f_) and non-floral aboveground (DW_nf_) tissues, and harvest index (HI) of *Cannabis sativa* ‘Meridian’ plants grown under light emitting diodes (LEDs) for 45 days under average canopy-level photosynthetic photon densities (PPFD) of either 600, 800, or 1,000 μmol m^–2^ s^–1^ for 12 h day^–1^ or PPFD of 600 μmol m^–2^ s^–1^ plus ultraviolet (UV, 280–400 nm) of either 12 h day^–1^ of 50 μmol m^–2^ s^–1^ from LEDs with peak wavelength of 385 nm for 45 days (UVA) or 5 h day^–1^ of 3 μmol m^–2^ s^–1^ of wideband ultraviolet fluorescent lighting for the last 20 days of the flowering cycle (UVA + UVB).

Biomass parameter	Treatment					Significance^x^
	1,000	800	600	UVA	UVA + UVB	
FW_f_ (g)	218±3.8^z^*a*^y^	191 ± 5.1^ab^	143 ± 10.0^c^	162 ± 6.7^bc^	151 ± 7.5^c^	*
FW_nf_ (g)	108 ± 4.8	102 ± 1.7	84 ± 10.3	91 ± 5.3	92 ± 0.8	ns
DW_f_ (g)	44.7 ± 0.94^a^	37.2 ± 1.71^ab^	27.6 ± 2.22^c^	31.3 ± 1.49^bc^	29.3 ± 1.40^bc^	*
DW_nf_ (g)	28.3 ± 1.24	25.8 ± 0.49	20.4 ± 2.70	22.2 ± 1.49	22.4 ± 0.25	ns
HI^w^	0.61 ± 0.015	0.58 ± 0.007	0.58 ± 0.011	0.58 ± 0.007	0.57 ± 0.009	ns

^w^Harvest index was calculated using: HI = DW_f_/(DW_f_ + DW_nf_).

^x^ns, not significant; *, significant at *P* ≤ 0.05.

^y^For each row, means followed by the same lowercase letter are not different at *P* ≤ 0.05 according to Tukey’s honestly significant difference test.

^z^Data are means ± SE (*n* = 3). Harvest index is the proportion of total aboveground DW that is comprised of inflorescence biomass.

**FIGURE 3:**
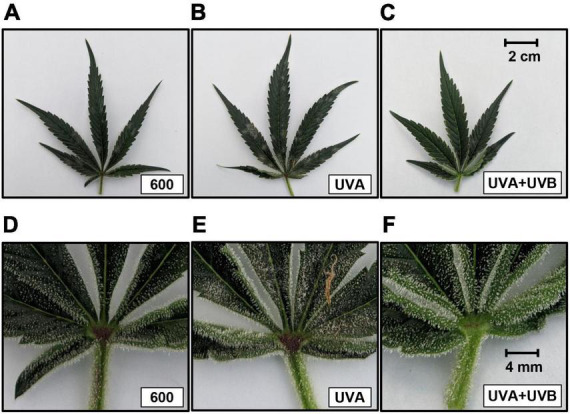
Images of upper canopy leaves from representative plants in the 600 μmol m^–2^ s^–1^ (control) **(A,D)**, UVA **(B,E)**, and UVA + UVB **(C,F)** treatments. The images in the upper row are entire leaves. The scale bar in the upper right corner of 3c is 2.0 cm and is the same size for all images in the upper row. The images in the lower row are 5× magnifications of the foliar portions proximate to the petiole of the respective upper row images. The scale bar in the upper right corner of 3f is 4 mm and is the same size for all images in the lower row.

At the time of harvest, the apical inflorescences in the light intensity treatments all had similar appearance, inferring that the lighting intensities did not affect the rate of inflorescence maturation. Typical for this genotype, CBD concentrations were below detection limits in both floral tissues in all treatments. There were no intensity or spectrum treatment effects on the detected cannabinoid concentrations of floral (Table 3) tissues. Along with CBD, neither CBN nor D8THC were detected in the floral tissues from any treatment. There were also no intensity or spectrum treatment effects on either individual terpenes (data not shown) or total terpenes concentrations in floral tissues (Table 3). Foliar THC concentrations were higher in the UVA + UVB vs. the control and UVA treatments, but this did not affect the composite T-THC concentrations due to relatively high proportions of THCA in all treatments (Table 4). It did appear that the UVA + UVB treatment reduced the severity of powdery mildew on upper canopy leaves and may have enhanced the production of foliar trichomes, particularly in basal areas of the leaflets proximate to the petiole (Figure 3).

**TABLE 3:** Cannabinoid and total terpene content (mg g^–1^) of dry composite inflorescence samples of *Cannabis sativa* ‘Meridian’ plants grown under light emitting diodes (LEDs) for 45 days with average canopy-level photosynthetic photon densities (PPFD) of either 600, 800, or 1,000 μmol m^–2^ s^–1^ for 12 h day^–1^ or PPFD of 600 μmol m^–2^ s^–1^ plus ultraviolet (UV, 280–400 nm) from either UVA (12 h day^–1^ of 50 μmol m^–2^ s^–1^ from LEDs with peak wavelength of 385 nm for 45 days) or UVA + UVB [5 h day^–1^ of 3 μmol m^–2^ s^–1^ of wideband UV fluorescent lighting for the last 20 days of the flowering cycle (UVA+UVB)].

Secondary metabolite^z^	Treatment					Significance^x^
	1,000	800	600	UVA	UVA + UVB	
CBG	0.67 ± 0.059^y^	0.68 ± 0.010	0.61 ± 0.040	0.61 ± 0.041	0.74 ± 0.027	ns^w^
CBGA	14 ± 1.1	12 ± 0.3	11 ± 0.1	11 ± 0.7	11 ± 0.5	ns
T-CBG	13 ± 1.0	11 ± 0.3	10 ± 0.1	10 ± 0.6	10 ± 0.3	ns
THC	6.4 ± 0.27	6.3 ± 0.47	7.5 ± 0.16	7.5 ± 0.28	7.5 ± 0.86	ns
THCA	249 ± 6.9	226 ± 7.5	225 ± 5.4	216 ± 9.1	222 ± 3.6	ns
T-THC	225 ± 5.8	205 ± 6.6	205 ± 4.6	197 ± 8.3	202 ± 4.3	ns
Total terpenes	17 ± 0.2	19 ± 0.8	19 ± 0.6	18 ± 0.8	17 ± 0.7	ns

^w^Not significant at *P* ≤ 0.05.

^x^Treatment effects for each parameter were evaluated at *P* ≤ 0.05 according to Tukey’s honestly significant difference test.

^y^Data are means ± SE (*n* = 3).

^z^CBG, cannabigerol; CBGA, cannabigerolic acid; T-CBG, total equivalent cannabigerol; THC, Δ^9^-tetrahydrocannabinol; THCA, Δ^9^-tetrahydrocannabinolic acid; T-THC, total equivalent Δ^9^-tetrahydrocannabinol. Total terpenes is the sum of the concentrations of individual terpenes. Both acid and neutral forms of cannabidiol (CBD), cannabinol (CBN) and Δ^8^-tetrahydrocannabinol (D8THC) were below the 0.5 mg g^–1^ limit of detection.

**TABLE 4:** Cannabinoid content of dry foliar (‘sugar leaves’) of *Cannabis sativa* ‘Meridian’ plants grown under three lighting treatments: 12 h day^–1^ of 600 μmol m^–2^ s^–1^ of photosynthetically active radiation for 45 days (Control), 12 h day^–1^ of 600 μmol m^–2^ s^–1^ of PAR plus an additional 12 h day^–1^ of 50 μmol m^–2^ s^–1^ of ultraviolet (UV, 280–400 nm) either from LEDs (385 nm peak) for 45 days (UVA) and 12 h day^–1^ of 600 μmol m^–2^ s^–1^ of PAR for 45 days plus an additional 3 μmol m^–2^ s^–1^ of wideband UV fluorescent lighting for the last 20 days of the flowering cycle (UVA + UVB).

Secondary metabolite^z^	Content in dry “sugar” leaves (mg g^–1^)			Significance^w^
	Control	UVA	UVA + UVB	
CBC	0.19 ± 0.013^y^	0.19 ± 0.026	0.20 ± 0.011	ns
CBGA	1.1 ± 0.17	1.3 ± 0.11	1.3 ± 1.10	ns
T-CBG	1.0 ± 0.14	1.1 ± 0.10	1.1 ± 0.06	ns
THC	2.8 ± 0.20b^x^	2.8 ± 0.12^b^	3.7 ± 0.14^a^	*
THCA	30 ± 3.2	29 ± 1.6	32 ± 1.2	ns
T-THC	29 ± 2.9	28 ± 1.5	32 ± 1.1	ns

^w^ns, not significant; *, significant at *P* ≤ 0.05.

^x^For each row, means followed by the same letter are not different at *P* ≤ 0.05 according to Tukey’s honestly significant difference test.

^y^Data are means ± SE (*n* = 3).

^z^CBC, cannabichromene; CBGA, cannabigerolic acid; T-CBG, total equivalent cannabigerol; THC, Δ^9^-tetrahydrocannabinol; THCA, Δ^9^-tetrahydrocannabinolic acid; T-THC, total equivalent Δ^9^-tetrahydrocannabinol. Cannabigerol (CBG) and both acid and neutral forms of cannabidiol (CBD), cannabinol (CBN) and Δ^8^-tetrahydrocannabinol (D8THC) were below the 0.5 mgg^–1^ limit of detection.

## Discussion

Two of the dominant phytogenic factors that affect profitability in commercial drug-type cannabis production are marketable yield (i.e., the mass of mature, unfertilized female inflorescences) and the secondary metabolite composition (i.e., concentrations of cannabinoids and terpenes) in these marketable tissues. A primary objective of this study was to explore proof of concept for the potential for UV radiation treatments for increasing cannabinoid content, particularly THC, in a modern indoor-grown cannabis genotype with a relatively high THC content. The genotype used in this study was typical of Type-I (i.e., drug-type) cannabis (de Meijer et al., 1992); with >20% THC (i.e., ≥200 mg g^–1^) and low CBD in the inflorescence tissue. A low amount of cannabigerol (CBG, the chemical precursor to both THC and CBD) was detected. The ratio of T-THC to total CBG (T-CBG) was ≥17, indicating that the majority of the cannabinoid production in this cultivar had reached the targeted end point. There was also no cannabinol (CBN) – a natural THC breakdown product – detected in any sample (data not shown) which, along with the high ratio of T-THC to T-CBG, indicated that the plants were near peak maturity at harvest (Aizpurua-Olaizola et al., 2016).

### Increasing light intensity proportionally increases yield

Many (interrelated) environmental parameters can be optimized in order to maximize yields, including temperature, humidity, CO_2_ concentration, and fertility. However, in indoor cultivation environments, LI is one of the most prominent and expensive input parameters under the complete control of the cultivator (Mills, 2012). The optimum LI in a given production scenario will depend on many economic factors, but the responses of modern cannabis genotypes’ yield and secondary metabolite composition to LI are key input factors that can only be elucidated experimentally. Compared with the 600 μmol m^–2^ s^–1^ treatment, increasing the PAR exposure by 1.6 times (i.e., 1,000 μmol m^–2^ s^–1^) increased inflorescence dry yield by the same magnitude. This implies that, for yield responses in this trial, the cannabis plants growing under 1,000 μmol m^–2^ s^–1^ were still on the linear phase of the light response curve (i.e., still operating at maximum quantum efficiency). This is also supported by the linear yield responses to increasing LI up to 1,800 μmol m^–2^ s^–1^ reported in Rodriguez-Morrison et al. (2021a). A linear yield response to a range of LIs that exceeds normal production levels (Potter and Duncombe, 2012), confers a relatively reliable and easily interpreted basic model for how cannabis yield responds to changes in LI. For example, a simple regression of the mean DW_f_ at the three tested PPFD levels (i.e., for calculating slope) in the present study predicts that every additional 100 μmol m^–2^ s^–1^ of daily PAR will increase yield by 4.6 g/plant (i.e., 51 g m^–2^ at the present study’s planting density).

Although not statistically significant in this trial, the harvest index (HI, i.e., proportion of marketable aboveground biomass) also rose with increasing LI, similar to Rodriguez-Morrison et al. (2021a). This trend has also been observed in other species, but the rate of increase in cannabis HI was approximately 4-fold higher than in indoor-grown wheat (Bugbee and Salisbury, 1988) over a similar LI range. This serves as further evidence of cannabis’ enormous phenotypic plasticity in response to LI. Higher HI could enhance harvest efficiency by reducing the non-marketable proportion of the biomass, all of which needs to be removed at harvest and disposed (EMCDDA, 2012). Further, since inflorescence tissues have substantially higher cannabinoid contents than other aboveground tissues (Richins et al., 2018), plants that produce proportionally higher inflorescence biomass under higher LI may also increase overall cannabinoid yield. While this was not evaluated in the present study, it is also likely that the increased HI was associated with larger inflorescences and increased floral density, such as reported in Rodriguez-Morrison et al. (2021a). These attributes are generally highly valued by the industry, particularly when the crop production is targeted toward the dry inflorescence market which presently accounts for over 60% of total cannabis product sales in Canada (Health Canada, 2021).

### Potential costs and benefits of increasing light levels in commercial production

The slope of the yield response curve relates to a crop’s phenotypic plasticity to respond to changes in environmental inputs – light intensity in this case – which will of course vary by genotype and production environment (Backer et al., 2019; Zheng and Llewellyn, 2022). Nevertheless, using the present study’s genotype as a proxy, one can estimate the payoff associated with increases in LI. For example, increasing the PPFD by 100 μmol m^–2^ s^–1^ increases the total light integral over 45 days by 195 mol m^–2^. The estimated increase in yield of 51 g m^–2^ for 195 mol m^–2^ of additional lighting corresponds to a light use efficiency of 0.26 g mol^–1^. The energy cost for additional lighting relies heavily on fixture efficacy, light distribution, and local cost of electricity. Most modern horticultural LED fixtures have efficacy values exceeding 2.5 μmol J^–1^ (i.e., 9 mol kWh^–1^) (Design Lights Consortium, 2022). If electricity cost was 0.10 $CAD/kWh, the estimated energy cost in this scenario would be approximately 0.042 $CAD/g or an energy use efficiency of approximately 2.4 g kWh^–1^ (for lighting). The increases in fixture efficacy in modern horticultural LEDs (Kusuma et al., 2020) may explain two-times higher estimated energy use efficiency in the present study vs. estimates from scientific studies in past decades (EMCDDA, 2012). At the current wholesale price for dried inflorescence of 4.00 $CAD/g (Cannabis Benchmarks, 2022), the added electricity cost of increasing yield by increasing LI comprises only ≈1% of the total price, and therefore may make economic sense. However, the costs of additional lighting infrastructure and ancillary costs such as heat management and higher crop production inputs must also be considered when assessing the potential profitability associated with any lighting strategy.

### Light intensity did not substantively affect chemical composition

The lack of LI effects on inflorescence cannabinoid content was consistent with other studies (Vanhove et al., 2011; Potter and Duncombe, 2012; Rodriguez-Morrison et al., 2021a). The lack of LI effects on inflorescence terpene content was also consistent with Rodriguez-Morrison et al. (2021a). Overall, both cannabinoid and terpene yield (i.e., g/plant or g m^–2^) increased concurrently with increasing inflorescence DW, which may be important for processing cannabis extracts. While many factors are involved in evaluating profitability of adopting a specific production practice, raising canopy-level LI may be an economically feasible way to increase inflorescence and secondary metabolite yield – but not concentration – in indoor cannabis production.

### Secondary metabolite content was unaffected by ultraviolet radiation

Ultraviolet radiation radiation can invoke both eustress and distress responses in plants, depending on many intrinsic (e.g., genotype and ontological stage) and extrinsic factors (e.g., UV intensity, duration, and spectrum, PAR intensity). The major modes of action of UV radiation on plants are through photoreceptor-mediated responses (e.g., UVR8) and the production of reactive oxygen species (ROS), which can cause cellular damage and influence gene expression (Jansen et al., 1998; Hideg et al., 2013). One common response to UV exposure is the production of photoprotective compounds, particularly in epidermal regions, to diminish UV penetration deeper into plant tissues (Frohnmeyer and Staiger, 2003; Huché-Thélier et al., 2016). In indoor cannabis production, the primary goal of exposing flowering plants to UV radiation is to upregulate the production of cannabinoids which are predominantly synthesized in the copious glandular trichomes that are found on and around inflorescence tissues (Potter, 2014) and which have UV-photoprotective properties (Hazekamp et al., 2005). Since this trial investigated a THC-dominant cultivar, it would be expected that any UV-induced upregulation of cannabinoid synthesis would result in substantially higher THC concentrations in the inflorescence and surrounding foliar (i.e., sugar leaves) tissues.

Since there were no yield reductions, there was little evidence of spectrum-induced distress in either of the UV treatments. While we did not quantify trichomes, we noted that there appeared to be higher trichome density on the sugar leaves in the UVA + UBV vs. the UVA and control treatments, particularly in areas proximate to the petioles. This observed increase in trichome density may explain the ≈30% higher foliar THC content and trends (not statistically significant) of ≈10% higher THCA and T-THC content in the UVA + UBV treatment vs. control. However, since foliar cannabinoid content was much lower than inflorescence tissues due to lower trichome density (Small, 2017), these tissues are of relatively low value in commercial indoor cannabis production and are often discarded (Potter, 2014). Therefore, from a production perspective, the minor increases in foliar cannabinoid concentration under UV exposure were probably not commercially relevant. Since none of the inflorescence cannabinoid levels were affected by the UV treatments, the eustress levels of UV radiation in this study did not have substantial effects on the secondary metabolite composition of the cannabis genotype used in this investigation. It is possible that the relatively low cannabinoid content of genotypes used in prior studies (e.g., Pate, 1983; Lydon et al., 1987) had conferred a relatively greater potential for stress-induced cannabinoid upregulation than in modern genotypes with much higher cannabinoid content (Dujourdy and Besacier, 2017). Further, within a given genotype’s overall genetic potential for producing various cannabinoids, there may be a higher likelihood for a plant to upregulate the production of one metabolite over another if both are normally present at relatively high concentrations. Therefore, in contrast with the present study, a genotype with a characteristically more balanced ratio of THC to CBD (i.e., type II) may show higher plasticity toward modifying the cannabinoid metabolome under UV eustress conditions. This may be particularly relevant to THC and CBD since they have the same biochemical precursor. However, this was not found to be the case in the two genotypes investigated in Rodriguez-Morrison et al. (2021b), however, their UV exposure protocols had only detrimental impacts on plant health, yield, and secondary metabolite composition. The lack of UV treatment effects in the type I chemotype grown in the present study may also indicate that this cultivar was already functioning near its maximum genetic capacity for the production of THC, thus UV exposure had no promotion effects on cannabinoid composition.

Unlike the PPFD parameter in assimilation lighting, where all photons within the 400–700 nm waveband are given equal weight, plant responses to UV can be very dynamic across the UVB and UVA wavebands. According to Flint and Caldwell (2003), the efficacy of the UV spectrum for inducing plant responses decreases by more than a factor of 200 as wavelength increases from 280 to 400 nm; with almost half of this reduction occurring in the narrow 300–310 nm range. Therefore, the spectrum distribution of a UV treatment is an extremely important descriptive parameter for any UV treatment protocol. For example, the raw flux density in the UVA treatment was ≈15 times higher but the BSWF-converted daily UV dose was ≈10% lower than in the UVA + UVB treatment. In prior studies, UV exposure levels were often described according to their daily “dose”, normally expressed in units of radiant flux density (e.g., kJ m^–2^), however, incomplete spectrum distribution information makes it difficult to compare UV treatment levels between studies (e.g., Fairbairn and Liebmann, 1974). We are aware of only two prior cannabis studies that provided quantitative data on UV exposure levels (summarized in Table 1). No raw flux density or spectral data was provided in Lydon et al. (1987) but their reported maximum BSWF-adjusted daily UV doses were approximately 30% higher than both treatments in the present study and are comparable to the maximum UV dose in Rodriguez-Morrison et al. (2021b). Due to the different UV exposure periods, the total BSWF-adjusted UV doses were lowest in the present study, moderate in Lydon et al. (1987) and highest in Rodriguez-Morrison et al. (2021b).

### Considerations for future research on ultraviolet radiation in cannabis production

Since any level of UV exposure in Rodriguez-Morrison et al. (2021b) was detrimental to cannabis growth and yield, it is probable that the spectrum of their UV treatments was predominantly injurious to cannabis, irrespective of the intensity. That the UV exposure was invoked concurrently with the transition to the flower-prompting 12-h PAR photoperiod may have exacerbated the deleterious effects. This contrasts with the Lydon et al. (1987) study whose plants were intentionally acclimated to UV (at an unknown level) for 30 days prior to the commencement of the experiment. There was no UV acclimation period in the present study, but the exposure period for the UVA + UVB treatment was initiated much later in flowering cycle than the UVA treatment. Even though the BSWF-corrected daily UV dose was higher in the UVA + UVB treatment, the total dose was less than half of the UVA treatment. Further, both total UV doses in the present study were considerably lower than the maximum levels used in Lydon et al. (1987) and Rodriguez-Morrison et al. (2021b), especially given the higher levels of PAR. Since the UV intensity relative to PAR can affect the magnitude of plant responses to UV (Kotilainen et al., 2018), it may be important to also consider the UV exposure level relative to the PAR intensity, especially when growing cannabis under higher PPFDs. A good target might be to match the photon flux ratio of UV to PAR in sunlight, which is about 1:20 (Nikiforos et al., 2011), while also being mindful that ≤5% of the solar UV is in the UVB range (i.e., ≤315 nm). Further, despite the low proportion of UVB in total solar UV, this proportion increases to ≈35% when applying the BSWF adjustment to the solar UV waveband (data not shown).

Given the negative effects of UV exposure in Rodriguez-Morrison et al. (2021b), positive effects in Lydon et al. (1987), and negligible effects in the present study relative to their total doses, it is still possible that there is a UV exposure protocol that provokes eustress over distress responses in some cannabis chemotypes. Despite the similar daily UV doses among these studies, the differences in daily UV photoperiod, number of days of UV exposure before harvest, total UV dose, cannabis chemotypes, and potential effects of PAR exposure level (Table 1) all illustrate the variety of potential UV exposure protocols. There are four major factors that need to be considered for the development of UV exposure protocol in cannabis cultivation: spectrum, intensity, daily duration, and the total exposure period relative to harvest. The time of day, relative to the PAR photoperiod, for UV treatments may also be an important consideration. The present study had day-long exposures to UVA but the UVA + UVB treatment was only provided during the last 5 h of the PAR photoperiod, during which workers were prohibited from being present in the research area. Similarly, the UV treatments in Rodriguez-Morrison et al. (2021b) were only provided after the end of the normal workday, however, the UV exposures in Lydon et al. (1987) spanned the midday period. Midday UV exposures may more closely match the daily dynamics of solar UV levels, however, the practical UV exposure period in indoor cannabis cultivation systems may primarily be constrained by need to minimize the risks of employee exposure to UV.

While there are still myriad combinations of cannabis genotype, spectrum, and UV dose paradigms yet to be studied, there is still little evidence that UV exposure has commercially-relevant benefits to either cannabis yield or quality in indoor production systems. Based on the combined results of this trial and Rodriguez-Morrison et al. (2021b), we recommend investigating the use of longer-wavelength UVB LEDs (e.g., peak wavelength of ≈310 nm) and UV exposures focused on the pre-harvest periods of the short-photoperiod flowering stage (i.e., after vegetative growth slows). Along with inflorescence yield and secondary metabolite composition, investigators should characterize temporal morphological effects of UV exposure on developing inflorescence and associated tissues (e.g., density and composition of glandular trichomes).

## Conclusion

Cannabis proliferates at very high canopy LIs in indoor production environments. The increasing inflorescence (and associated cannabinoid) yield responses to high LI in this trial clearly shows the benefits to maximizing canopy-level PPFD within the economical constraints imposed by other production logistics (including input costs). Conversely, we saw no commercially-relevant benefits to exposing cannabis plants to UV radiation. Given the myriad potential UV exposure algorithms (i.e., combinations of spectrum, intensity, and temporal application strategies) more research is needed to determine if and how UV exposure in indoor cannabis production may be a commercially-relevant production tool and elucidate appropriate treatment protocols for commercial applications.

## Data availability statement

The raw data supporting the conclusions of this article will be made available by the authors, without undue reservation.

## Author contributions

DL, SG, and EF performed the experiment and collected the data. DL analyzed the data and wrote the manuscript draft. YZ, SG, EF, SD, and AJ revised the manuscript. All authors contributed to the experimental design and approved the final manuscript.

## Abbreviations

BSWF: biological spectral weighting function for plant growth
CBC: cannabichromene
CBD: cannabidiol
CBG: cannabigerol
CBN: cannabinol
DW_f_: dry weight of inflorescence
DW_nf_: dry weight of non-floral aboveground tissues
FW_f_: fresh weight of inflorescence
FW_nf_: fresh weight of non-floral aboveground tissues
HI: harvest index
LI: light intensity
PAR: photosynthetically active radiation
PFD: photon flux density
PPFD: photosynthetic photon flux density
Δ^9^-THC: Δ ^9^-tetrahydrocannabinol
UV: ultraviolet (100–400 nm)
UVA: ultraviolet-A (315–400 nm)
UVB: ultraviolet-B (280–315 nm).

## References

[B1] Aizpurua-Olaizola O. Soydaner U. Öztürk E. Schibano D. Simsir Y. Navarro P. (2016). Evolution of the cannabinoid and terpene content during the growth of *Cannabis sativa* plants from different chemotypes. *J. Nat. Prod.* 79 324–331. 10.1021/acs.jnatprod.5b00949 26836472

[B2] Backer R. Schwinghamer T. Rosenbaum P. McCarty V. Eichhorn Bilodeau S. Lyu D. (2019). Closing the yield gap for cannabis: A meta-analysis of factors determining cannabis yield. *Front. Plant Sci.* 10:495. 10.3389/fpls.2019.00495 31068957 PMC6491815

[B3] Bilodeau S. Wu B.-S. Rufyikiri A.-S. MacPherson S. Lefsrud M. (2019). An update on plant photobiology and implications for cannabis production. *Front. Plant Sci.* 10:296. 10.3389/fpls.2019.00296 31001288 PMC6455078

[B4] Bugbee B. Salisbury F. (1988). Exploring the limits of crop productivity. *Plant Physiol.* 88 869–878. 11537442 10.1104/pp.88.3.869 PMC1055676

[B5] Caldwell M. M. (1971). Solar UV irradiation and the growth and development of higher plants. *Photophysiology* 6, 131–177. 10.1016/b978-0-12-282606-1.50010-6

[B6] Cannabis Benchmarks (2022). *Canada Cannabis Spot Index.* Available online at: https://www.cannabisbenchmarks.com/report-category/canada (accessed April 12, 2022).

[B7] Cash C. Cunnane K. Fan C. Romero-Sandoval E. (2020). Mapping cannabis potency in medical and recreational programs in the United States. *PLoS One* 15:e0230167. 10.1371/journal.pone.0230167 32214334 PMC7098613

[B8] Chandra S. Lata H. Khan I. Elsohly M. (2008). Photosynthetic response of *Cannabis sativa* L. to variations in photosynthetic photon flux densities, temperature and CO2 conditions. *Physiol. Mol. Biol. Plants* 14 299–306. 10.1007/s12298-008-0027-x 23572895 PMC3550641

[B9] de Backer B. Debrus B. Lebrun P. Theunis L. Dubois N. Decock L. (2009). Innovative development and validation of an HPLC/DAD method for the qualitative and quantitative determination of major cannabinoids in cannabis plant material. *J. Chromatography* 877 4115–4124. 10.1016/j.jchromb.2009.11.004 19932642

[B10] de Meijer E. van der Kamp H. van Eeuwijk F. (1992). Characterisation of cannabis accessions with regard to cannabinoid content in relation to other plant characters. *Euphytica* 62 187–200.

[B11] Design Lights Consortium (2022). *Qualified Product List.* Available online at: https://www.designlights.org/horticultural-lighting/search/ (accessed April 12, 2022).

[B12] Dujourdy L. Besacier F. (2017). A study of cannabis potency in France over a 25 years period (1992-2016). *Forensic Sci. Int.* 272 72–80. 10.1016/j.forsciint.2017.01.007 28122324

[B13] EMCDDA (2012). *Cannabis Production and Markets in Europe.* Luxembourg: Publications Office of the European Union, 1-268 10.2810/52425:

[B14] Fairbairn J. W. Liebmann J. A. (1974). The cannabinoid content of *Cannabis sativa* L. grown in England. *J. Pharm Pharmacol.* 26 413–419. 4154985 10.1111/j.2042-7158.1974.tb09306.x

[B15] Flint S. Caldwell M. (1996). Scaling plant ultraviolet spectral responses from laboratory action spectra to field spectral weighting factors. *J. Plant Physiol.* 148 107–114. 10.1016/S0176-1617(96)80301-4

[B16] Flint S. Caldwell M. (2003). A biological spectral weighting function for ozone depletion research with higher plants. *Physiol. Plant.* 117 137–144. 10.1034/j.1399-3054.2003.1170117.x

[B17] Frohnmeyer H. Staiger D. (2003). Ultraviolet-B radiation-mediated responses in plants: Balancing damage and protection. *Plant Physiol.* 133 1420–1428. 10.1104/pp.103.030049 14681524 PMC1540342

[B18] Gill S. S. Anjum N. A. Gill R. Jha M. Tuteja N. (2015). DNA damage and repair in plants under ultraviolet and ionizing radiations. *ScientificWorldJournal.* 2015:250158. 25729769 10.1155/2015/250158 PMC4333283

[B19] Green A. Sawada T. Shettle E. (1974). The middle ultraviolet reaching ground. *Photochem. Photobiol.* 19 251–259. 10.1111/j.1751-1097.1974.tb06508.x

[B20] Hazekamp A. Peltenburg A. Verpoorte R. Giroud C. (2005). Chromatographic and spectroscopic data of cannabinoids from *Cannabis sativa* L. *J. Liquid Chromatography Related Technol.* 28 2361–2382.

[B21] Health Canada (2021). *Cannabis Market Data: Overview.* Available online at: https://www.canada.ca/en/health-canada/services/drugs-medication/cannabis/research-data/market.html (accessed April 14, 2022).

[B22] Hideg E. Jansen M. Strid Å (2013). UV-B exposure, ROS, and stress: Inseparable companions or loosely linked associates? *Trends Plant Sci.* 18 107–115. 23084465 10.1016/j.tplants.2012.09.003

[B23] Huché-Thélier L. Crespel L. le Gourrierec J. Morel P. Sakr S. Leduc N. (2016). Light signaling and plant responses to blue and UV radiations—perspectives for applications in horticulture. *Environ. Exp. Bot.* 121 22–38.

[B24] Jansen M. A. K. Gaba V. Greenberg B. M. (1998). Higher plants and UV-B radiation: Balancing damage, repair and acclimation. *Trends Plant Sci.* 3 131–135.

[B25] Jikomes N. Zoorob M. (2018). The cannabinoid content of legal cannabis in Washington state varies systematically across testing facilities and popular consumer products. *Sci. Rep.* 8:4519. 29540728 10.1038/s41598-018-22755-2 PMC5852027

[B26] Kotilainen T. Robson T. M. Hernández R. (2018). Light quality characterization under climate screens and shade nets for controlled-environment agriculture. *PLoS One* 13:e0199628. 10.1371/journal.pone.0199628 29940006 PMC6016941

[B27] Krizek D. T. (2004). Influence of PAR and UV-A in determining plant sensitivity and photomorphogenic responses to UV-B radiation. *Photochem. Photobiol.* 79 307–315. 10.1562/2004-01-27-ir.1 15137505

[B28] Kusuma P. Pattison P. M. Bugbee B. (2020). From physics to fixtures to food: Current and potential LED efficacy. *Hortic. Res.* 7:56. 10.1038/s41438-020-0283-7 32257242 PMC7105460

[B29] Layton C. Aubin A. J. (2019). *UPLC Separation for the Analysis of Cannabinoid Content in Cannabis Flowers and Extracts.* Available online at: https://www.waters.com/webassets/cms/library/docs/720006509en.pdf (accessed April 14, 2022).

[B30] Livingston S. J. Quilichini T. D. Booth J. K. Wong D. C. J. Rensing K. H. Laflamme−Yonkman J. (2020). Cannabis glandular trichomes alter morphology and metabolite content during flower maturation. *Plant J.* 101 37–56. 10.1111/tpj.14516 31469934

[B31] Lydon J. Teramura A. H. Coffman C. B. (1987). UV-B radiation effects on photosynthesis, growth and cannabinoid production of two *Cannabis sativa* chemotypes. *Photochem. Photobiol.* 46 201–206. 10.1111/j.1751-1097.1987.tb04757.x 3628508

[B32] Magagnini G. Grassi G. Kotiranta S. (2018). The effect of light spectrum on the morphology and cannabinoid content of *Cannabis sativa* L. *Med. Cannabis Cannabinoids* 1 19–27. 10.1159/000489030 34676318 PMC8489345

[B33] Mah J. J. Llewellyn D. Zheng Y. (2019). *Protocol for Converting Spectrometer Radiometric Data to Photon Flux Units, TechNote 001-2019 [Microsoft Excel Spreadsheet].* Guelph, ON: University of Guelph.

[B34] Mills E. (2012). The carbon footprint of indoor cannabis production. *Energy Policy* 46 58–67.

[B35] Nikiforos K. Eduardo R. Robert S. (2011). The value of the ratio of UVA to UVB in sunlight. *Photochem. Photobiol.* 87 1474–1475. 10.1111/j.1751-1097.2011.00980.x 21770951

[B36] Pate D. W. (1983). Possible role of ultraviolet radiation in evolution of cannabis chemotypes. *Econ. Bot.* 37 396–405.

[B37] Pate D. W. (1994). Chemical ecology of cannabis. *J. Int. Hemp Assoc.* 2 32–37.

[B38] Potter D. (2009). *The Propagation, Characterisation and Optimisation of Cannabis sativa L. As a Phytopharmaceutical.* London: King’s College London.

[B39] Potter D. (2014). A review of the cultivation and processing of cannabis (*Cannabis sativa* L.) for production of prescription medicines in the UK. *Drug Test. Anal.* 6 31–38. 10.1002/dta.1531 24115748

[B40] Potter D. Duncombe P. (2012). The effect of electrical lighting power and irradiance on indoor-grown cannabis potency and yield. *J. Forensic Sci.* 57 618–622. 10.1111/j.1556-4029.2011.02024.x 22211717

[B41] Richins R. D. Rodriguez-Uribe L. Lowe K. Ferral R. O’Connell M. A. (2018). Accumulation of bioactive metabolites in cultivated medical cannabis. *PLoS One* 13:e0201119. 10.1371/journal.pone.0201119 30036388 PMC6056047

[B42] Rodriguez-Morrison V. Llewellyn D. Zheng Y. (2021a). Cannabis yield, potency, and leaf photosynthesis respond differently to increasing light levels in an indoor environment. *Front. Plant Sci.* 12:646020. 10.3389/fpls.2021.646020 34046049 PMC8144505

[B43] Rodriguez-Morrison V. Llewellyn D. Zheng Y. (2021b). Cannabis inflorescence yield and cannabinoid concentration are not increased with exposure to short-wavelength ultraviolet-B radiation. *Front. Plant Sci.* 12:725078. 10.3389/fpls.2021.725078 34795683 PMC8593374

[B44] Small E. (2017). *Cannabis: A Complete Guide.* Boca Raton, FL: CRC Press.

[B45] Vanhove W. Van Damme P. Meert N. (2011). Factors determining yield and quality of illicit indoor cannabis (*Cannabis* spp.) production. *Forensic Sci. Int.* 212 158–163. 10.1016/j.forsciint.2011.06.006 21737218

[B46] Zheng Y. Llewellyn D. (2022). “Lighting and CO2 in cannabis production,” in *Handbook of Cannabis Production in Controlled Environments*, ed. Zheng Y. (Boca Raton, Fl: CRC Press, Taylor & Francis), 163–188.

